# *MiR137* is an androgen regulated repressor of an extended network of transcriptional coregulators

**DOI:** 10.18632/oncotarget.5958

**Published:** 2015-10-05

**Authors:** Emeli M. Nilsson, Kristian B. Laursen, Jonathan Whitchurch, Andrew McWilliam, Niels Ødum, Jenny L. Persson, David M. Heery, Lorraine J. Gudas, Nigel P. Mongan

**Affiliations:** ^1^ Faculty of Medicine and Health Sciences, School of Veterinary Medicine and Science, University of Nottingham, United Kingdom; ^2^ Department of Pharmacology, Weill Cornell Medical College, New York, NY, USA; ^3^ School of Pharmacy, University of Nottingham, United Kingdom; ^4^ Department of Immunology and Microbiology, University of Copenhagen, Copenhagen, Denmark; ^5^ Clinical Research Center, Lund University, Malmö, Sweden

**Keywords:** epigenetic, prostate, nuclear receptor, metastases, Gleason

## Abstract

Androgens and the androgen receptor (AR) play crucial roles in male development and the pathogenesis and progression of prostate cancer (PCa). The AR functions as a ligand dependent transcription factor which recruits multiple enzymatically distinct epigenetic coregulators to facilitate transcriptional regulation in response to androgens. Over-expression of AR coregulators is implicated in cancer. We have shown that over-expression of KDM1A, an AR coregulator, contributes to PCa recurrence by promoting VEGFA expression. However the mechanism(s) whereby AR coregulators are increased in PCa remain poorly understood. In this study we show that the microRNA *hsa-miR-137 (miR137)* tumor suppressor regulates expression of an extended network of transcriptional coregulators including *KDM1A/LSD1/AOF1, KDM2A/JHDM1A/FBXL11, KDM4A/JMJD2A, KDM5B JARID1B/PLU1, KDM7A/JHDM1D/PHF8, MED1/TRAP220/DRIP205* and *NCoA2/SRC2/TIF2*. We show that expression of *miR137* is increased by androgen in LnCaP androgen PCa responsive cells and that the *miR137* locus is epigenetically silenced in androgen LnCaP:C4-2 and PC3 independent PCa cells. In addition, we found that restoration of *miR137* expression down-regulates expression of *VEGFA*, an AR target gene, which suggests a role of *miR137* loss also in cancer angiogenesis. Finally we show functional inhibition of *miR137* function enhanced androgen induction of *PSA/KLK3* expression. Our data indicate that *miR137* functions as an androgen regulated suppressor of androgen signaling by modulating expression of an extended network of transcriptional coregulators. Therefore, we propose that epigenetic silencing of *miR137* is an important event in promoting androgen signaling during prostate carcinogenesis and progression.

## INTRODUCTION

Epigenetic silencing of tumor suppressor microRNAs (miRs) has recently emerged as an important mechanism of carcinogenesis [1]. MiRs are endogenous mediators of RNA silencing which function to attenuate transcriptional and translational outputs [2]. Each miR can functionally interfere with multiple target mRNAs *in vivo*, and thus a single miR has the potential to down-regulate an extended network of transcriptional targets. Conversely, the epigenetic loss of a specific miR would permit increased expression of multiple target genes. Numerous examples of such tumor suppressor miRs have been described [reviewed in 1, 3]. Epigenetic silencing of *hsa-miR-137* (*miR137*) has recently emerged as an important event in neuroblastoma [4], glioblastoma [5], colorectal [6, 7], breast [8], head and neck [9, 10] and bladder [11] cancers. Known, validated targets for *miR137* include the cell cycle regulator *Cdc42* [7], estrogen related receptor *ERRα/NR3B1* [12], p160 nuclear receptor coactivators [13] and the lysine specific demethylase 1 (*KDM1A, LSD1*), a transcriptional coregulator [6] which is over-expressed in many cancers [14 and references therein]. Loss of *miR137* expression is associated with increased cell proliferation and invasion, consistent with a role in metastatic disease [6].

KDM1A plays important roles in androgen receptor (AR) signaling [15, 16] and is over-expressed in recurrent prostate cancer (PCa) [14, 17]. The AR is a member of the ligand dependent transcription factor superfamily of nuclear receptors and mediates the transcriptional actions of the androgens, testosterone, and dihydrotestosterone. The AR itself plays an essential role both in male development [18] and prostate [19] and other cancers [20]. Nuclear receptors, including the AR, recruit multiple enzymatically diverse transcriptional coregulators in response to agonist activation [21 and references therein]. These transcriptional coregulators are essential mediators of the epigenetic regulation of transcription by modulating histone modifications, including lysine methylation and acetylation [reviewed in 22, 23]. These coregulators mediate the transcriptional activities of other nuclear receptors, most notably the estrogen receptors [24] and other transcription factors [23]. Aberrant expression and function of transcriptional coregulators are implicated in numerous human malignancies, including PCa [14, 23, 25-29].

We [14] and others [15-17] have reported that expression of *KDM1A* is increased in PCa and is associated with poorer outcomes. We have also shown that *KDM1A* contributes to poorer prostate outcomes by promoting pro-androgenic and pro-angiogenic pathways [14]. However understanding of the mechanism(s) contributing to increased expression of *KDM1A* and other coregulators [30] in PCa remains incomplete. In this report we first sought to determine whether specific microRNAs, including *miR137,* which are expressed in normal prostate cells but lost in PCa cells, function as repressors of *KDM1A*. Our analysis revealed that *miR137* targets an extended network of transcriptional coregulators, including *KDM1A/LSD1/AOF1, KDM2A/JHDM1A/FBXL11, KDM4A/JMJD2A, KDM5B/JARID1B/PLU1, KDM7A/JHDM1D/PHF8, MED1/TRAP220/DRIP205* and *NCoA2/SRC2/TIF2.* Ectopic restoration of *miR137* expression decreases expression of these transcriptional coregulators. We show that restoration of *miR137* function is associated with a decrease in *VEGFA* expression which we have previously shown to be positively regulated by the AR-KDM1A complex [14]. We show that expression of *miR137* is increased by androgen in androgen-responsive cells. Finally, we show that functional inhibition of *miR137* enhances androgen signaling. Our data indicates that the epigenetic status of the *miR137* locus influences expression of an extended network of transcriptional coregulators and that *miR137* functions as an androgen regulated suppressor of epigenetic coregulators. We therefore propose that epigenetic silencing of *miR137* is an important event in promoting androgen signaling during prostate carcinogenesis and progression.

## RESULTS

### Expression of *miR137* is epigenetically silenced by DNA methylation in prostate cancer cells

**Figure 1:**
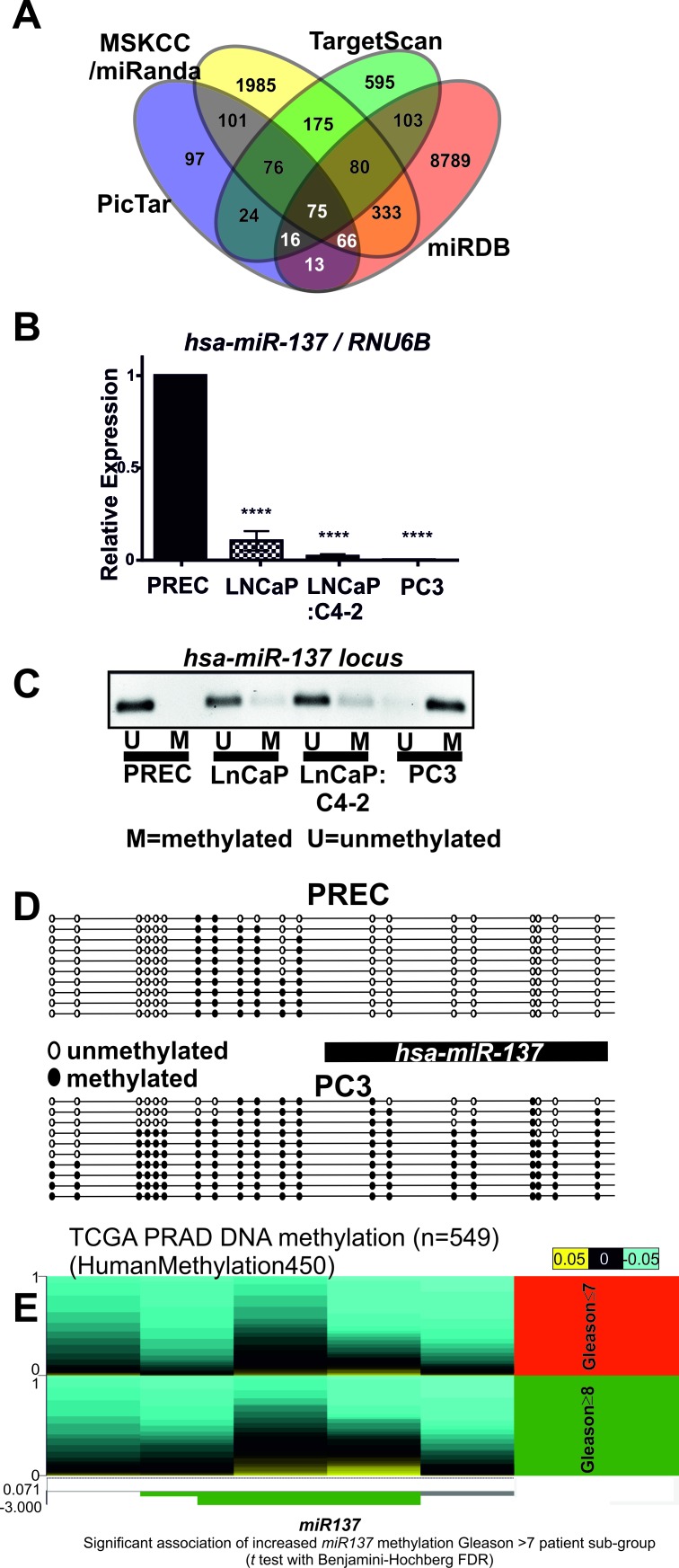
**A. Venn diagram of putative *miR137* target genes identified by four different tools**. **B.** Quantitative RTPCR was used to measure *miR137* expression in prostate cells. Normal prostate epithelial cells (PREC) exhibited the most abundant *miR137* expression as compared to LnCaP, LnCaP:C4-2 and PC3 PCa cells. **C.** Genomic DNA was isolated from prostate epithelial cells (PREC) and PCa cell lines, LNCaP, LNCaP:C4-2 and PC3 cells and subjected to bisulfite modification. The location of the *miR137* transcript relative to the CpGs analyzed is indicated. Methylation specific PCR was used to assess methylation a crucial CpG island. The *miR137* locus was unmethylated in normal PREC, but hemi-methylated in LNCaP and LNCaP:C4-2 cells. The *miR137* locus in PC3 was methylated. **D.** BSP primers were used to amplify the entire CpG island of the *miR137* locus in PREC and PC3 cells. The *miR137* locus was unmethylated in normal PREC, but densely methylated in PC3 PCa cells. Black circles indicate methylated CpGs, whereas white circles indicate unmethylated CpGs. The data represent a minimum of 10 independent alleles from each respective cell line. Consistent with the literature, methylation of the *miR137* locus in PCa cells correlates with lower *miR137* expression as compared with the unmethylated *miR137* locus in normal PREC. **E.** We used the cancer genome atlas prostate adenocarcinoma (PRAD) dataset DNA methylation dataset to correlate methylation of the *miR137* locus and Gleason grade. Increased DNA methylation (indicated in yellow) at the cg05423529, cg14783814 and cg22333214 locations in the *miR137* locus is significantly associated (*t* test with Benjamini-Hochberg FDR) with Gleason grades ≥8 patient sub-group (indicated in green, N-196) as compared to patients with Gleason grades ≤7 (indicated in red, *n* = 323)

Expression of *KDM1A* is increased in many solid tumors and leukemias [14 and references therein]. However the mechanisms whereby *KDM1A* expression is increased remain poorly understood. One potential mechanism relates to the loss of a repressor microRNA [31]. We therefore used the PicTar [32], Targetscan [33], miRDB [34] and miRanda [35] microRNA target prediction tools and identified *miR137* as a potential regulator of *KDM1A* (Figure 1A, Supplemental Table 1). *MiR137* was also recently shown to regulate *KDM1A* in colon cancer [6] and neuroblastoma [4]. MicroRNAs are known to regulate expression of multiple targets which are often functionally related. For this reason we next examined whether *miR137* also regulated expression of genes functionally related to *KDM1A*. Our analysis identified *KDM2A, KDM4A, KDM5B, KDM7A, MED1, CBP,* and *SUZ12* as potential *miR137* targets in PCa. By real time qPCR we detected abundant expression of *miR137* in normal prostate epithelial cells (PREC), but reduced *miR137* expression in LnCaP (representative of localized PCa) and absent *miR137* expression in LnCaP:C4-2 (a bone metastatic derivative of the parental LnCaP cell line) and PC3 (representative of aggressive androgen independent metastatic PCa) (Figure 1B). We next used bisulfite sequencing PCR (BSP) to determine the DNA methylation status of *miR137* locus in PREC and LNCaP. We confirmed these findings by completing methylation specific PCR (MSP) in PREC, LnCaP, LnCaP:C4-2 and PC3 cells. MSP and BSP indicate extensive methylation of the *miR137* locus in PCa cell lines (Figure 1C, 1D). We next assessed the DNA methylation status of the *miR137* locus in publically available patient genomic datasets [36] and the cancer genome atlas prostate adenocarcinoma (PRAD) DNA methylation dataset. The *miR137* locus harbors significantly higher methylation in tumor tissue as compared to non-malignant tissue in a variety of human cancer types, including PCa (Supplemental Figure 1). Indeed increased *miR137* methylation correlates with increasing PCa Gleason grade (Figure 1E). We have shown that expression of *miR137* was significantly lower in all PCa cells relative to normal prostate epithelial cells (Figure 1B). Consistent with this, our analysis of a published PCa patient transcriptomic cohort, shows that median *miR137* expression is lower in PCa patients who experience PCa recurrence (Supplemental Figure 2A), indicating epigenetic loss of *miR137* expression is associated with PCa recurrence [37].

### An extended network of transcriptional coregulators are novel *miR137* targets *in vitro*

**Figure 2:**
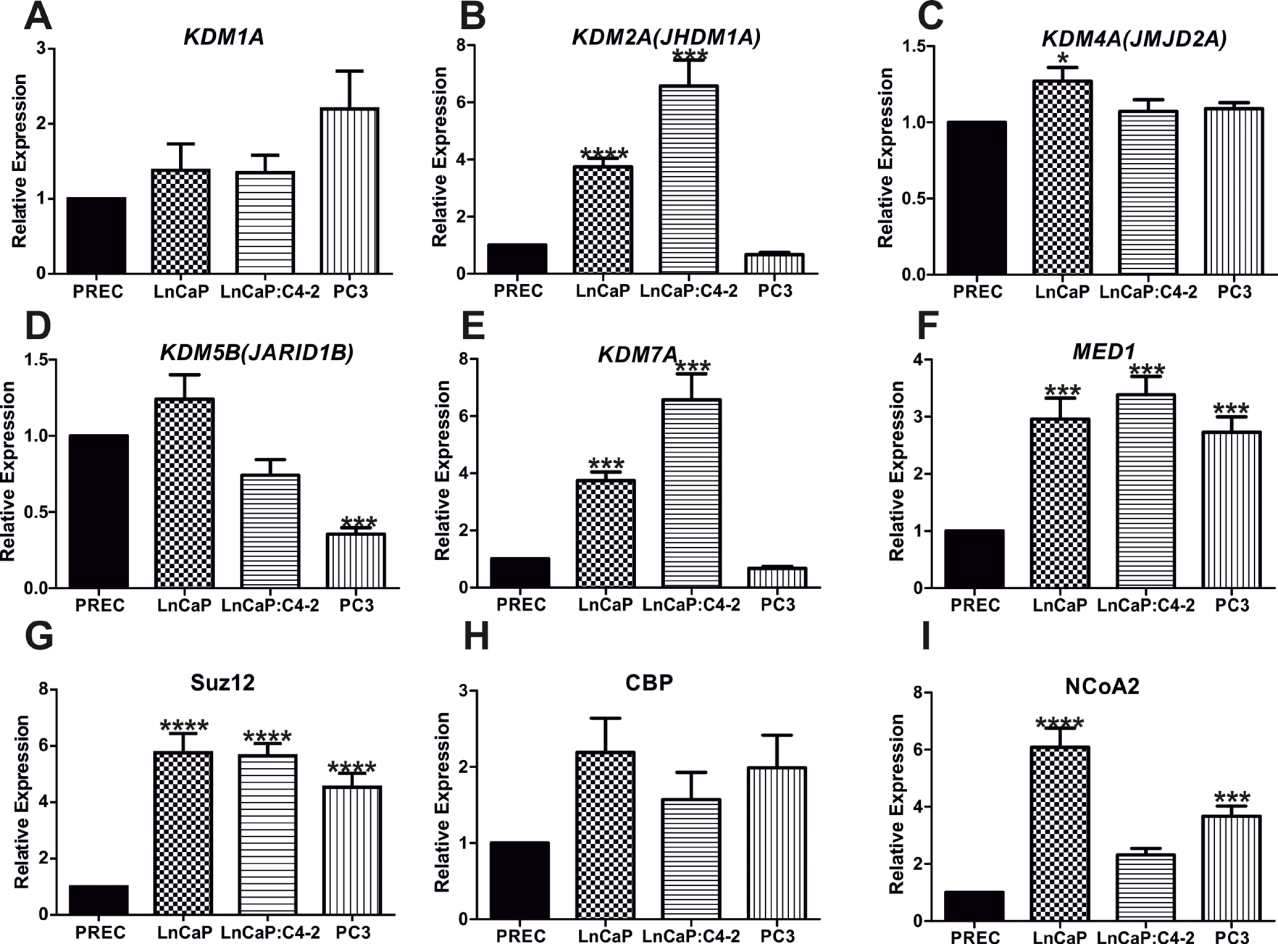
Identification and validation of *miR137* target genes Quantitative reverse transcriptase PCR was used to compare mRNA expression of putative *miR137* targets in non-malignant prostate epithelial cells (PREC), androgen responsive (LNCaP) and (LNCaP:C4-2 and PC3) hormone refractory PCa cells. Bars = mean ±SEM. * = *p* < 0.05; **p < 0.005,*** = *p* < 0.001, **** = *p* < 0.0001 by ANOVA with Bonferroni's post hoc test for multiple comparisons. Expression of all putative targets examined was similar or significantly higher in LnCaP and LnCAP:C4-2 PCa cell lines as compared to non-malignant PREC. Bars represent mean ±SEM of biological triplicate experiments.

**Figure 3:**
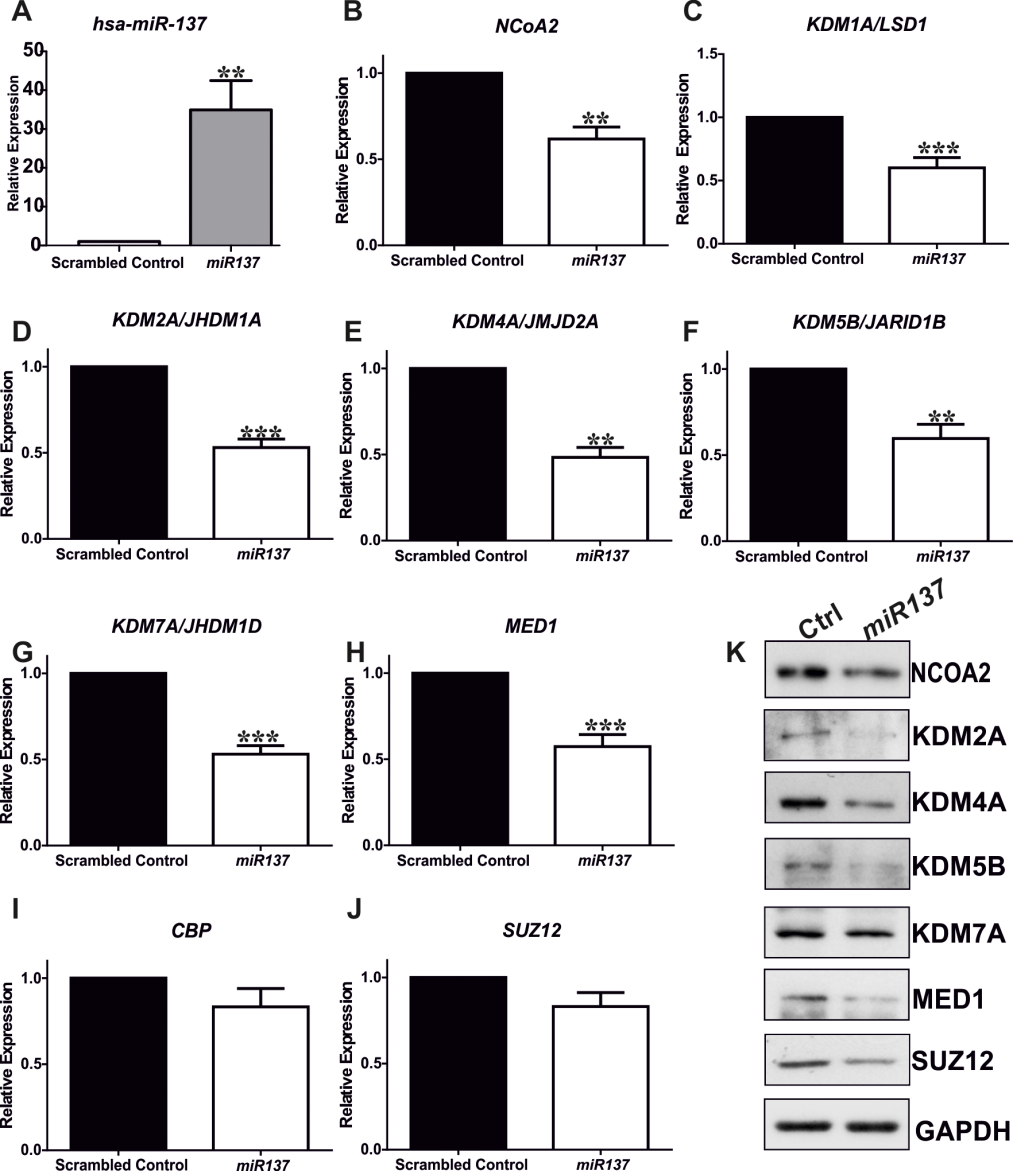
Quantitative RTPCR indicates ectopic over-expression of *miR137* in PC3 cells induces decreases in mRNA levels of *KDM1A/LDS1, NCoA2/SRC2, KDM2A, KDM4A, KDM5B, KDM7A,* and *MED1* A.-H. although the expression of CBP and SUZ12 was not significantly reduced I.-J. Bars represent mean ±SEM of triplicate experiments conducted on at least two independent occasions. * = *p* < 0.05; **p < 0.005,*** = *p* < 0.001, **** = p < 0.0001 by ANOVA with Bonferroni's post hoc test for multiple comparisons. Western blot analyses on extracts from PC3 cells expressing miR137 or scramble controls confirmed the reduced protein levels including in the case of SUZ12 (**K.**). Thus SUZ12 protein was decreased in PC3 cells over-expressing miR137, suggesting potential post-translational regulation of SUZ12 protein levels by miR137. qRTPCRs and western analyses were completed on a minimum of three biological replicates.

Novel putative *miR137* targets were prioritized for experimental validation if they were identified by a minimum of two tools, had a transcription coregulator-related function, and were expressed in PCa cells (Supplemental Table 1). To this end we examined mRNA expression of the putative *miR137* targets identified here, *KDM1A, KDM2A, KDM2A, KDM5B, KDM7A, CBP, SUZ12, MED1,* and *NCoA2* in normal and malignant PCa cells (Figure 2A-2I). Interestingly, the expression of all putative *miR137* targets tested was similar or elevated in *AR* expressing LnCaP and LnCaP:C4-2 PCa cells as compared to normal prostate epithelial cells. Having confirmed the expression of these genes in PCa cells, we next determined the effects of ectopic over-expression of *miR137* on the mRNA and protein levels of *KDM1A, KDM2A, KDM2A, KDM5B, KDM7A, CBP, SUZ12, MED1* and *NCoA2.* We over-expressed *miR137* and scrambled control non-targeting miRNA in PC3 cells which lack endogenous *miR137* but which express the validated *miR137* target *KDM1A* (Figure 2A). Expression of *NCOA2* and *KDM1A* was reduced following *miR137* over-expression (Figure 3B, 3C). Quantitative RTPCR and western blot analyses indicated that expression levels of *NCoA2, KDM2A, KDM4A, KDM5B, KDM7A* and *MED1* mRNA and protein (Figure 2) were decreased following over-expression of *miR137.* Expression of *CBP* transcript levels was unaffected by *miR137* (Figure 3I). Although the decrease in *SUZ12* did not reach statistical significance (Figure 3J), interestingly SUZ12 protein levels were decreased by *miR137* expression (Figure 3K). Previous studies have indicated *NCOA2* is regulated by *miR137* in other cancer types [13, 38]. Therefore we included NCOA2 both as a positive control in our studies and to confirm that this regulation is conserved in prostate cancer. GAPDH was used as a loading control.

**Figure 4:**
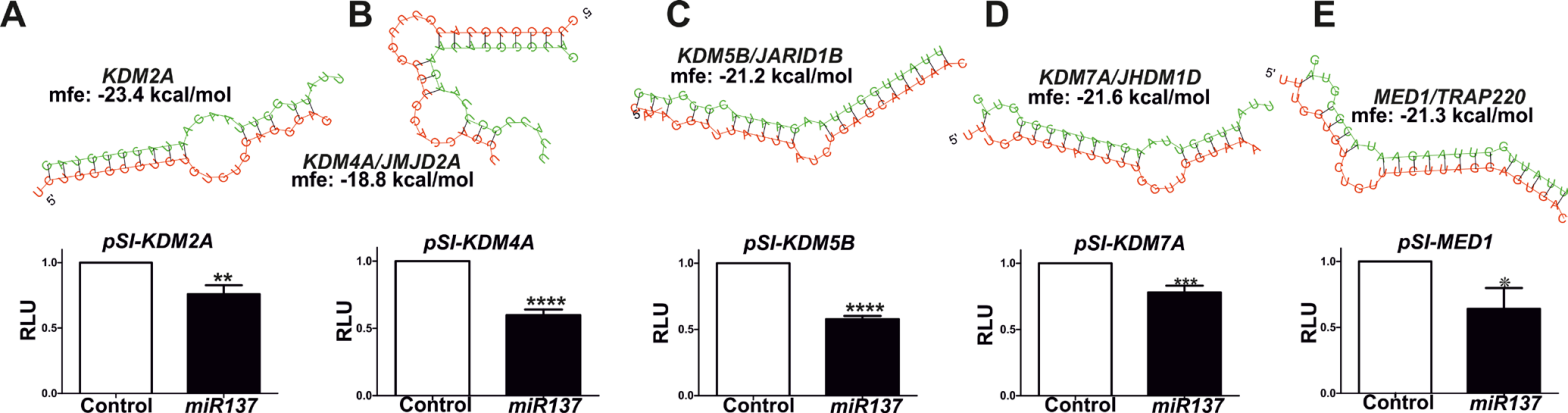
The RNAhybrid algorithm was used to determine the predicted hybridization of *miR137* (green) and the 3′UTRs of the novel putative *miR137* targets as indicated, and luciferase reporter assays used to confirm target specificity **A.,** *KDM2A* **B.**, *KDM4A* **C.**, *KDM5B* **D.**, *KMD7A* and **E.**, *MED1*. Luciferase assays were performed in PC3 cells transfected with either parental psiCHECK2 or psiCHECK2-3′UTR constructs for each putative *miR137* target and either a scrambled control or *miR137* expression construct. Bars represent mean ±SEM of minimum of three biological replicate experiments conducted on at least three independent occasions (*n* = 6-9). * = *p* < 0.05; **p < 0.005,*** = *p* < 0.001, **** = *p* < 0.0001 by *t* test.

To confirm these findings RNAhybrid [39] was used to calculate the minimum free energy for hybridization of *miR137* with target UTRs where we had evidence indicating that *miR137* decreases both mRNA and protein expression. We compared the calculated minimum free energy for known validated *miR137* targets *KDM1A* (−19.4 kcal/mol) and *NCoA2* (−19.9 kcal/mol). To test if *KDM2A, KDM4A, KDM5B, KDM7A* and *MED1* are direct targets of *miR137* we generated luciferase reporter constructs using the psiCHECK2 vector containing the predicted *miR137* target sequences identified within the 3′UTR of each putative novel target (Figure 4A-4E). Each reporter was cotransfected into PC3 cells with either the *miR137* or *miR*-scrambled control expression construct. Over-expression of *miR137* had no effect on luciferase reporter activity of the parental psiCHECK2 vector (data not shown), whereas over-expression of *miR137* caused a significant decreases in the relative luciferase reporter activity for each of the psiCHECK2 constructs harboring the putative 3′UTR target sequences for each gene, as compared to cells transfected with the scrambled miR negative control (Figure 4). This suggests that *KDM2A, KDM4A, KDM5B, KDM7A* and *MED1* are direct targets of *miR137.*

### Regulation of *miR137* expression in prostate cancer cells

**Figure 5:**
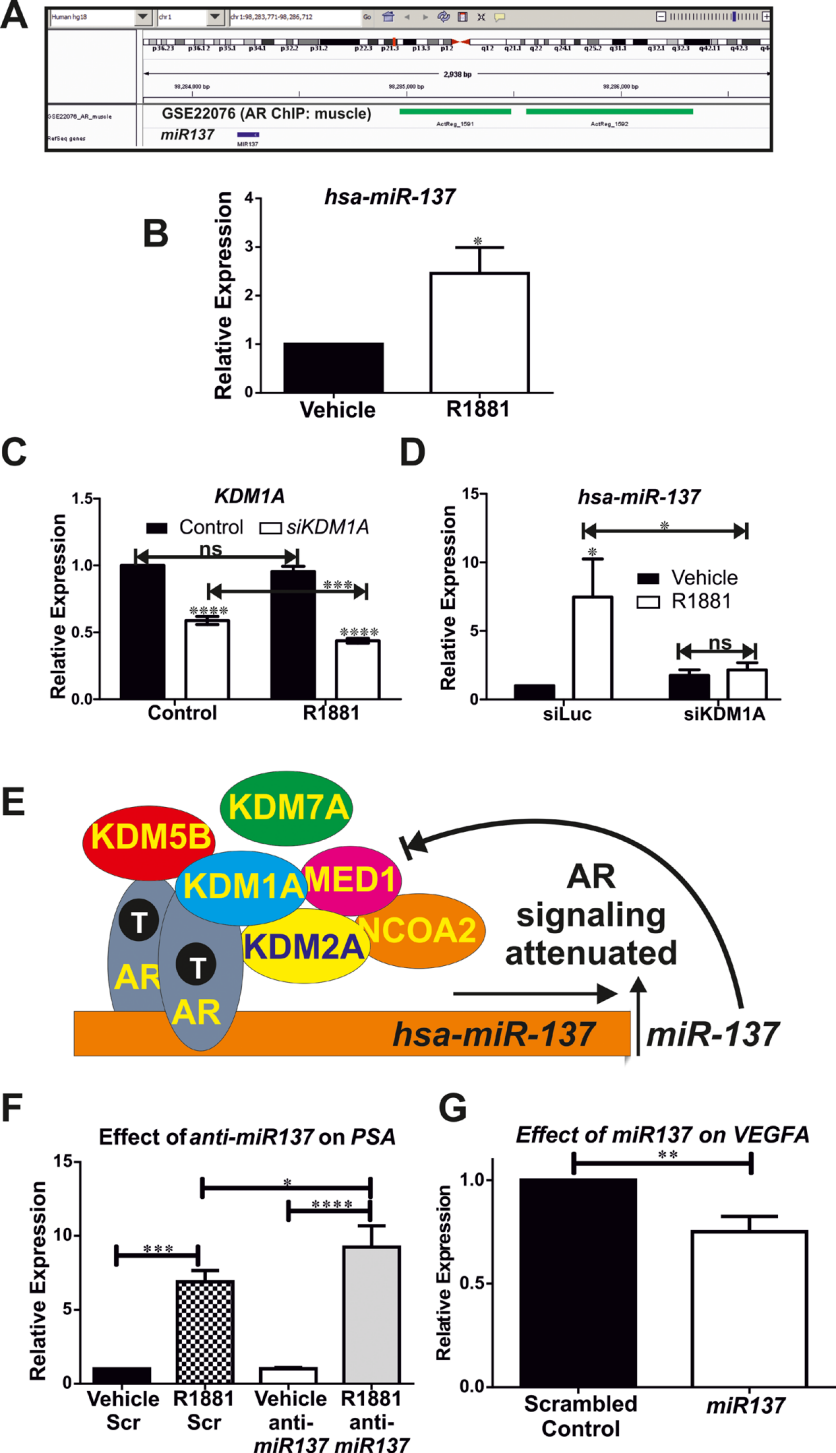
**A. Genomewide chromatin immune-precipitation (ChIP) assays (GSE22076) determined the distribution of the AR in muscle cells [41] indicate recruitment of AR (indicated in green) to the *miR137* locus (indicated in blue). B.** Quantitative RTPCR indicates expression of *miR137* induced by androgen in LnCaP cells. **C.**, **D.** siRNA mediated functional depletion of *KDM1A* indicates androgen induced expression of *miR137* requires KDM1A. There is a significant reduction in *KDM1A* expression in LNCaP treated with both *siKDM1A* with androgen as compared to LNCaP treated with *siKDM1A* and vehicle. This suggests that androgen induction of *miR137* augments siRNA mediated depletion of *KDM1A*. Data shown is the mean±SEM of a minimum of five biological independent experiments analyzed in triplicate. **E.** We propose a model whereby androgens, acting via the AR-coregulator complex induce expression of *miR137* which in turn acts to reduce coregulator levels and attenuate androgen transcriptional activation. The AR is known to interact with KDM1A, KDM2A, KDM5B, NCOA2 and MED1 whereas AR interaction with KDM7A has not been reported to date. **F.** Consistent with this model, quantitative RTPCR indicates a synthetic *miR137* antagomiR significantly enhances androgen induced transcription of *PSA/KLK3* in LnCaP cells. AntiMiR experiments were conducted on a minimum of six biological replicates analyzed in technical triplicates. **G.** Conversely ectopic expression of *miR137* reduces expression of the pro-angiogenic *KDM1A* target gene in the PC3 cell model of castrate resistant PCa. Data shown is the mean±SEM of a minimum of three independent experiments analyzed in triplicate. * = *p* < 0.05; **p < 0.005,*** = *p* < 0.001, **** = *p* < 0.0001

We next assessed the relationship between androgen and *miR137* expression. To this end, we used the integrated genome viewer [40] and the appropriate genome build to interrogate publically available AR genomewide chromatin immune-precipitation (ChIP) (GSE22076, GSE28219, GSE48308, GSE14092/97) datasets and identified AR recruitment to the *miR137* locus in androgen responsive, non-malignant muscle cells [41] (Figure 5A). Consistent with this, androgen treatment of androgen responsive LNCaP cells induced *miR137* expression (Figure 5B). We and others have previously shown that *KDM1A* is a crucial component of androgen induced and AR-mediated transcription in PCa cells [14-17, 42, 43]. We therefore tested whether KDM1A is involved in androgen regulation of *miR137* expression. To this end we used siRNA to functionally deplete *KDM1A* expression in LNCaP cells (Figure 5C) as previously described [14]. We found that KDM1A is required for androgen-induced *miR137* expression (Figure 5C, 5D).

### Effect of *miR137* expression on androgen target gene expression in prostate cancer cells

Collectively our data support a rheostat function for *miR137* in the autoregulation of androgen signaling, as depicted in Figure 5E. In non-malignant androgen responsive cells, AR/cofactor complexes, including KDMs, drive hormone-dependent transcription of target gene networks. This response is attenuated by induction of *miR137* expression, which reduces levels of essential AR coregulators. In malignant androgen responsive cells, the *miR137* locus can be epigenetically silenced by DNA methylation, allowing a sustained androgen response to drive androgenic proliferation pathways. Consistent with this, alterations in expression of these coregulators are associated with poorer PCa outcomes (Supplemental Figure 2B, 2C). To further test this model of *miR137* as a negative regulator of androgen-dependent transcription, we assayed the effect of a synthetic *miR137* antagomiR on androgen-induced expression of the prostate specific antigen gene, *PSA/KLK3.* As shown in Figure 5F, androgen treatment of LnCaP cells stimulated a ∼6 fold induction in *PSA/KLK3* expression. However functional inhibition of *miR137* by transfection of *anti-miR137* in LnCaP cells resulted in a ∼9 fold androgen induction of *PSA/KLK3* expression. Moreover ectopic expression of *miR137* reduces expression of the *VEGFA* pro-angiogenic factor (Figure 5G) which we have previously shown to be regulated by KDM1A [14]. Taken together our results provide strong evidence for a central role of *miR137* as a rheostat controlling androgen response in prostate cells. Induction of *miR137* by AR executes a negative feedback loop by suppressing expression of essential AR coregulators, in particular the KDMs, as outlined here. Disruption of this pathway may therefore play a key role in disease progression in PCa.

## DISCUSSION

There is considerable interest in the potential clinical value of *miRs* in PCa [44]. However, the successful exploitation of *miRs* as clinical prognostic markers and their utilization as therapeutic targets will require comprehensive molecular characterization of the diverse effects of individual *miRs* in distinct cellular contexts. It was previously established that *miR137* negatively regulates many genes implicated in cancer including the transcriptional coregulators *KDM1A* [4, 6, 45] and *NCOA2* [13, 38], aurora kinase A (AURKA) [6], estrogen-related receptor *ERRα/NR3B1* [12] and constitutive androstane receptor (*CAR*/*NF1I3*)[46], the *CDC42* and *CDK6* cell cycle regulators [7, 47], the ubiquitin ligase *MIB1[48], COX2* [5], *paxillin* [49], chromosome segregation like-1 *CSE1L* gene [50], glioma pathogenesis-related protein 1 (also referred to as RTVP1) [51], the *EZH2* polycomb protein [52] and *CtBP1* [53]. Interestingly, loss or reduced expression of *miR137* has been reported in colo-rectal cancer [6], neuroblastoma [4], glioblastoma [51], head and neck [10], non-small cell lung [54, 55] and gastric [56] cancers. This loss or reduction in *miR137* expression is commonly attributable to epigenetic silencing by DNA methylation. In this study we show that the *miR137* locus is unmethylated in non-malignant PREC, but is increasingly methylated in more aggressive PCa cell lines (Figure 1C). Consistent with this, our analysis of the TCGA DNA methylation datasets confirms increased DNA methylation of the *miR137* locus in all cancers examined with the exception of thyroid carcinoma. Indeed increased methylation of *miR137* was found in higher grade PCas (Figure 1). Furthermore there is evidence in the TCGA prostate dataset presented in the cBioPortal that up to 10% of prostate tumors harbor heterozygous deletions encompassing the *miR137* locus [57]. Restoration of *miR137* expression inhibits proliferation and the metastatic potential of colo-rectal cancer [6, 7, 49] and glioblastoma [51] and sensitizes cancer cells to chemotherapies [46, 58]. Collectively these numerous studies support an important role for epigenetic silencing of *miR137* in carcinogenesis, resistance to apoptosis, promotion of metastases and mediating multiple mechanisms of therapeutic resistance. It is in this context that we have identified five nuclear receptor transcriptional coregulators as novel targets of *miR137.* Our work expands the known repertoire of validated *miR137* targets as we have identified an extended network of known and potential AR transcriptional coregulators as novel *miR137* targets.

The AR plays essential roles in carcinogenesis and metastases, most notably in prostate and bladder cancers [59]. For this reason, androgen deprivation therapies (ADTs) are commonly used to treat PCa. Although ADTs are often initially effective, a transition to a hormone refractory state in which androgen-targeted therapies are no longer effective appears inevitable even for next generation ADTs such as abiraterone and enzalutamide [as reviewed in 60]. Indeed, it is now understood that androgen signaling persists following the transition to the hormone refractory, castrate resistant state [19, 60, 61, 62]. Current ADTs directly target AR function by blocking androgen biosynthesis (gonadotrophin releasing hormone analogues), acting as AR selective antagonists (flutamide, bicalutamide, enzalutamide) or blocking intra-tumoral androgen biosynthesis (abiraterone). However men receiving ADT commonly progress within 18 months to a castration resistant state for which no curative therapy is available. Given the essential roles for androgen signaling in all PCa stages, there is an urgent need to identify novel components of the AR signaling network which may represent novel PCa therapeutic targets. AR coregulators are attractive therapeutic targets as the AR must recruit a large repertoire of enzymatically diverse transcriptional coregulators. Indeed many of these AR coregulators are implicated in PCa through diverse molecular mechanisms [63]. These include AR coregulators with intrinsic or recruited lysine acetyltransferase (KATs) activity such as the p160 (NCOA1, NCOA2, NCOA3) and p300/CBP coactivators [28, 63-69], lysine methyltransferases (KMTs) such as G9A and SYMD3 [70, 71], lysine demethylases (KDMs) such as KDM1A/LSD1 [16, 42], KDM4A/JMJD2A [72, 20], JMJD2C [15], JMJD2D [72], KDM5B/JARID1B [30] and the MED1 component of the mediator complex [73].

*MiR137* represses *KDM1A* [6] and *NCOA2* [38]. Although epigenetic loss of *miR137* contributes to increased *KDM1A* expression in colo-rectal [6] and oral [38] cancers respectively, the mechanisms resulting in increased *KDM1A* and NCOA2 in PCa cells are not well understood. *NCOA1, NCOA2* and *NCOA3* are *miR137* targets in prostate, breast and melanoma cancers and the *miR137* locus is silenced in PCa [13]. In our study we have confirmed that *miR137* is epigenetically silenced in pre-clinical PCa cell models of localized and castrate resistant cancer and demonstrate that increased *miR137* methylation correlates with increasing Gleason grade in a clinical dataset (Figure 1). Furthermore, we have shown that *miR137* is predicted to act as a repressor of an extended network of transcriptional coregulators, in addition to *KDM1A* (Supplemental Table, Figure 1). We first confirmed expression of the putative novel *miR137* targets (*KDM2A, KDM5B, KDM7A, CBP, SUZ12, MED1*) and the *KDM1A* and *NCoA2* validated *miR137 targets* in PCa cell lines (Figure 2). Ectopic expression of *miR137* in PC3 cells, which lack intrinsic *miR137* expression, results in decreased mRNA and protein levels of *KDM1A, KDM2A, KDM4A, KDM5B, KDM7A* and *MED1* but had no effect on *CBP* or *SUZ12* mRNA levels (Figure 3). However protein levels of SUZ12 were decreased, suggesting *miR137* interferes with SUZ12 protein levels through an alternative mechanism. Related to this, *miR137* is known to regulate the EZH2 component of the polycomb repressive complex 2 (PRC2) of which SUZ12 is also an integral component [52]. It is possible that *miR137* mediated depletion of EZH2 induces dissociation of PRC2 thereby contributing to decreased SUZ12 observed here. In addition we found that *miR137* repressed luciferase reporters containing the predicted *miR137* target sequences from the 3′UTR of *KDM2A, KDM4A, KDM5B, KDM7A and MED1.* Collectively, our data supports *miR137* as a negative regulator for the transcriptional *KDM2A, KDM4A, KDM5B, KDM7A* and *MED1* in PCa cells.

NCOA2, KDM1A, KDM4A, KDM5B and MED1 are AR coregulators implicated in prostate and other cancers [16, 30, 65, 73-78]. KDM1A promotes PCa recurrence by enhancing androgen induced pro-proliferative and pro-angiogenic transcription [14, 17]. Similarly, NCOA2, more commonly referred to as SRC2/TIF2 is a p160 AR coactivator implicated as an oncogene in prostate carcinogenesis and castrate resistance [74-78]. KDM2A/JHDM1A/FBXL11 acts on histone mono- and dimethylated histone H3K36 and is implicated in lung cancer [79] and the epigenetic silencing of chromatin [80]. The tri-methyl selective KDM4A/JMJD2A demethylase is an AR coregulator [72] expressed in PCa [14] and is a determinant of mTOR inhibitor sensitivity in non-small cell lung cancer patients [81]. Several studies have confirmed that the histone H3 lysine 4 selective demethylase, KDM5B interacts with AR, acts as an AR transcriptional coactivator, is increased in PCa and can act as an oncogene [30, 82 and references therein, 82-85]. KDM7A/JHDM1D/KIAA1718 is a dual-specificity demethylase which removes repressive marks on histone-H3, lysine 9 and 27 (H3K9me, H3K27me) [86, 87]. There is evidence that related members of this JHDM1-family of demethylases function as nuclear receptor transcriptional coregulators [88] and are implicated in PCa [76]. Given the ability of KDM7A to remove histone lysine methylation marks commonly associated with transcriptional repression (H3K9/K27) it is possible that KDM7A can also promote androgen induced transcription. However there is also evidence that in certain contexts increased JHDM1D can act as a tumor suppressor [89]. Further work is required to delineate the specific functions of these KDMs in PCa cells. KDMs are attractive therapeutic targets and the availability of next generation KDM selective inhibitors [43, 90, 91] raises the potential for development of novel therapies which can circumvent resistance to existing ADTs by targeting the KDM activities required for the functional AR-complex.

As little is known about the regulation of *miR137* in prostate cells, we interrogated publically available ChIP datasets and identified AR recruitment to the *miR137* locus in non-malignant cells (Figure 5A). Based on this finding we tested the potential for androgen regulation of *miR137* and found a significant induction of *miR137* expression in the androgen responsive LnCaP which possess hemi-methylated *miR137* loci (Figure 5B). Thus androgens induce a negative regulator of AR-associated coregulators. We next determined whether KDM1A participates in androgen regulation of *miR137* expression. To address this we used established siRNA methods to functionally deplete *KDM1A* in LnCaP cells (Figure 5C) [14]. Consistent with our previous result (Figure 5B), expression of *miR137* is increased by androgen, but this androgen induction of *miR137* requires KDM1A (Figure 5D). Furthermore, there is a significant reduction in *KDM1A* expression in LNCaP treated with both *siKDM1A* with androgen as compared to LNCaP treated with *siKDM1A* and vehicle. This suggests that androgen induction of *miR137* augments siRNA mediated depletion of *KDM1A* (Figure 5C).

Our study has identified a negative feedback mechanism whereby androgens can attenuate androgen responsivity by titrating the levels of important coregulators through expression of *miR137* (Figure 5). We hypothesized that impairment or loss of androgen induction of *miR137* due to aberrant methylation of the *miR137* locus would relieve this brake on coregulator levels and would amplify androgen signaling. To test this we used a synthetic *miR137* antagomiR to inhibit *miR137*. We found that functional interference of *miR137* in LnCaP cells (Figure 1B) induces a 9 fold androgen induction of *PSA/KLK3* expression, as compared to a 6-fold induction of *PSA/KLK3* expression obtained in androgen treated LnCaP cells transfected with a scrambled antagomiR (Figure 5F). Therefore, we show that loss of *miR137* promotes androgen induced transcription. Conversely, the ability of a single miR to interfere with expression of multiple target genes emphasizes the great potential in therapeutic targeting of miRs. Although significant pharmacologic barriers remain to the deployment of miRs as systemic cancer therapies, we tested the effect of restoration of *miR137* in the PC3 model of advanced castrate resistant PCa. We found that restoration of *miR137* expression decreases expression of the pro-angiogenic factor *VEGFA* (Figure 5G), a key mediator of PCa metastases and therapy resistance [59].

In conclusion, our data indicates that *miR137* executes an androgen driven feedback inhibition of multiple AR coregulators in normal prostate epithelia and early stage PCa. Conversely, *miR137* silencing in advanced disease promotes over-expression of these coregulators and in turn hyper-activation of AR signaling. The ability of *miR137* to modulate AR signaling supports *miR137* as a potential therapeutic target for preventing or delaying prostate carcinogenesis and progression. Future studies should also focus on the multiple epigenetic effects attributable to the loss of *miR137.* Our study further highlights the important role of reciprocal regulatory networks involving microRNAs, nuclear receptors and coregulators in PCa. Our data supports the development of new therapies targeting epigenetic coregulators as a mechanism to overcome resistance to ADTs which currently target the AR but not the associated coregulator components of the AR complex.

## MATERIALS AND METHODS

### Bioinformatics: miR target prediction, clinical correlation, protein network analysis and statistics

A survey of putative targets of *miR137* was conducted using PicTar [32], TargetScan [33], miRDB [34], and miRanda (microRNA.org) target prediction algorithms [35, 92]. RNAhybrid was used to determine the minimum free energy requried for hybridization of *miR137* and the predicted novel targets [39]. Candidates identified by a minimum of two prediction algorithms and with a known function in transcripion were selected for further analysis. The *MethHC* tool [36] was used to statistically compare methylation of the *miR137* locus in tumor and non-tumor tissue from the TCGA database. The UCSC cancer genome browser [93] was used to correlate *miR137* locus methylation with PCa recurrence. Statistical analysis was performed using GraphPad Prism 5.04 (GraphPad, La Jolla, CA). For qPCR and luciferase reporter experiments t-tests, one-way ANOVA with Dunnett's and/or Bonferroni post-test multiple comparison test was performed using GraphPad Prism*. P*-values < 0.05 were considered statistically significant with 95% confidence intervals.

### Cell culture, transfection, siRNA, androgen (R1881) treatments

Normal prostate epithelial cells (PREC) and PCa cell lines LNCaP, LNCaP:C4-2 and PC-3 cells were maintained as described previously [14, 94]. PREC were sourced from Lonza (Walkersville, MD), LnCaP and PC3 were purchased from ATCC. LnCaP:C4-2 were a generous gift from Dr. Doug Scherr, Department of Urology, Weill Cornell Medical College. Androgen treatment (R1881) and siRNA depletion of *KDM1A* and *luciferase* as negative control were conducted as previously described [14, 95]. PC3 cells were stably transfected with over-expression constructs for human *miR137* and scrambled controls (#HmiR0011-MR04 and #CmiR0001-MR04, GeneCopoeia, Inc.), using Lipofectamine 2000 (Invitrogen). Transfection efficiency was 80-90% as assessed by GFP expression and stable transfectants selected using puromycin (4μg/ml). Total RNA and protein were isolated from three independent stably transfected PC3 lines expressing *miR137* or the scrambled control.

### DNA methylation analysis, RNA extraction and Taqman real-time PCR

The methylation status of the *miR137* locus in PREC, LNCaP, LNCaP:C4-2 and PC-3 cells was determined by performing methylation specific analysis on bisulfite modified DNA essentially as previously described [6]. Genomic DNA (350ng) was subject to bisulfite conversion using the EZ-DNA Methylation Kit (#D5001; Zymo Research Corporation, Orange, CA) according to the manufacturer's instructions. The methylation status of the *miR137* locus was determined using bisulfite sequencing PCR (BSP) or methylation specific PCR (MSP) [6]. BSP products were cloned into pGEM-T (Promega, Southampton, UK), transformed into *SURE E.coli* competent cells (Stratagene/Agilent Technologies, Wokingham, UK). A minimum of 10 independent colonies were selected and plasmids containing the BSP inserts were sequenced directly to assess percentage methylation [96].

RNA (miRNA and total) was extracted using miRNeasy Mini kit (Qiagen, Crawley, UK) according to manufacturer's instructions. For miRNA quantitative reverse transcription polymerase chain reaction (qPCR) expression analysis, RNA (1μg) was reverse transcribed using the miScript reverse transcription kit (Qiagen, Crawley, UK) and miR-specific assays for human *miR137 (*MS00003486) and the *RNUD6* (218293) and/or *snoRD6* (MS00033705) normalization controls (Qiagen, Crawley, UK). For mRNA expression analysis, RNA (1μg) was reverse transcribed using qScript cDNA SuperMix (Quanta Biosciences, VWR, Lutterworth, UK) [14]. The resulting cDNA was used as a template for hydrolysis probe qPCR using the following gene specific hydrolysis probe assays (Invitrogen/Life Technologies/Thermo Scientific, Renfrew, UK): *KDM1A* (Hs01002741_m1), *KDM2A* (Hs00367034_m1), *KDM4A* (Hs00206360_m1), *KDM5B* (Hs00981910_m1), *KDM7A* (Hs01398501_m1), *MED1* (Hs01062349_m1), *NCoA2* (Hs00896114_m1), *CBP* (Hs00231733_m1), *SUZ12* (Hs00248742_m1), *VEGFA* (Hs00900055_m1) and *GAPDH* (Hs03929097_g1). All qPCR experiments were carried out on a minimum of three independent RNA isolations analyzed in triplicate.

### miR target validation

Luciferase reporter contructs were generated containing the predicted *miR137* target sequences from 3′-UTRs of each of the identified putative *miR137*-targets with a known role in transcription *(KDM1A, KDM2A, KDM4A, KDM5B, KDM7A, MED1,* and *NCoA2). NCoA2* has previously been shown to be a direct *miR137* target and was therefore included as a positive control target 3′-UTR. The predicted *miR137* target sequences identified within the 3′UTR of the putative novel targets were cloned into *XhoI* and *NotI* sites of the psiCHECK2­ luciferase reporter construct (Promega) using the primers indicated (Table 1). An empty psiCHECK2 vector was used as negative control. Plasmid DNA for over-expression constructs for human *miR137* and scrambled controls (#HmiR0011-MR04 and #CmiR0001-MR04, GeneCopoeia, Inc. Rockville, MD), psiCHECK2 negative control and psiCHECK2-3′UTR constructs for the identified targets were prepared using Prepease endotoxin free midiprep kit (USB). PC3 cells (1 × 10^5^), which lack endogenous *miR137* expression (Figure 1C), were plated in 24-well plates and allowed to attach for 24 hours prior to transfection. Cells were transfected with constructs expressing *miR137* or a scrambled control and either the psiCHECK2 empty vector control or psiCHECK2 containing the 3′UTR of the putative *miR137* targets of the genes identified. A total of 500ng DNA was used per well and cells were transfected using lipofectamine 2000 (Invitrogen/LifeTechnologies/ThermoScientific) according to the manufacturer's instructions. After 48 hours, cells were harvested and luciferase activity measured using the dual luciferase assay system (Promega) with a Turner TD-20/20 luminometer (Turner Designs, Sunnyvale, CA) as previously described [97]. Data were normalized to firefly luciferase. Luciferase experiments were conducted in triplicate and repeated on three independent occasions. The miR137 antagomiR (MIN0000429) was purchased from Qiagen and transfected into LNCaP cells using lipofectamine 2000 (Invitrogen/Life Technologies/ThermoScientific) according to the manufacturer's directions. Cells were grown at 37oC for 72 hours prior to RNA isolation and quantitative RTPCR as described earlier.

**Table 1:** 

Gene	Forward (5′-3′)	Reverse (5′-3′)
KDM2A	ccgctcgaggaagccctacagagttagggaatg	ataagaatgcggccgcagtttctttctaaggccagttaatg
KDM4A	ccgctcgagcccaggattggagggcttcacacc	ataagaatgcggccgccattttattgctaaggacaaggtgatgc
KDM5B	ccgctcgaggaaattccagtaaatcctcatttg	ataagaatgcggccgcaatcgctaaagcaccaacacac
KDM7A	ccgctcgaggtaagaacactgcccgaagaacag	ataagaatgcggccgcgaaaatacatcaagacactaccaac
MED1	ccgctcgaggtgcatgtatatgaagggctggg	ataagaatgcggccgcccatgactcaaacggacaactac

**Table 2:** 

Coregulator	Histone substrate	Non-histone substrates
KDM1A(LSD1/AOF1)	H3K4me2/1; H3K9me2/1	p53, E2F1, SNMT1
KDM2A (JHDM1A/FBXL11)	H3K36me2/1	p65, NF-kB
KDM4A (JMJD2A)	H3K9me3;H3K36me3, H1.4K26me3/2	
KDM5B (JARID1B)	H3K4me3/2	
KDM7A (JHDM1D/KIAA1718)	H3K9me2/1; H3K27me2/1	
MED1 (DRIP205/TRAP220)	histone acetylation via CBP/p300	
NCOA2 (SRC2/TIF2)	intrinsic histone acetylation and via CBP/p300	

### Western blotting

For protein expression analysis, after three days in culture PC-3-*miR137* and PC-3-*scrambled* control expressing cells were washed with PBS and lysed in SDS denaturing buffer (100 mM Tris-HCl pH 6.8, 4% SDS and 20% glycerol). The protein concentration in the cell lysates was quantified using the Bio-Rad DC Protein assay (Bio-Rad, Hercules, USA). Protein samples (10 μg) were boiled in loading buffer (83 mM Tris pH 8.8, 30% sucrose, 0.00083% bromophenol blue, 3% SDS and 8.3mM DTT) for 5 minutes, separated on 10% SDS-PAGE gels, and transferred to polyvinylidene difluoride membranes (Millipore Corporation, Bedford, USA). Membranes were blocked in 5% non-fat-milk for 1 hour and probed with primary antibodies against KDM2A/JHDM1A (Thermo Scientific; #PA5-11177; 5 μg/ml), KDM4A/JMJD2A (Cell Signaling Technology, Danvers, MA; #3393; 1:500), KDM5B/JARID1B (Cell Signaling; #3273; 1:1000), KDM7A/JHDM1D (Sigma-Aldrich, Gillingham, UK; #SAB2101190; 1 μg/ml), MED1/TRAP220 (R&D Systems, Abingdon, UK; #AF5520; 1 μg/ml), SUZ12 (Cell Signaling; #3737; 1:1000), NCoA2/TIF2 (BD Transduction Laboratories, Oxford, UK; #610985; 0.25 μg/ml), and GAPDH (Abcam, Cambridge, UK; #ab9484; 1 μg/ml) at 4°C overnight. Secondary antibodies used were donkey anti-goat, goat anti-mouse and goat anti-rabbit conjugated with horseradish peroxidase (Santa Cruz Biotechnology, Inc., Wembley, UK). Antibody binding was visualized using Amersham ECL Prime Western Blotting Detection Reagent (GE Healthcare, Amersham, UK). Western blots were performed on whole cell extracts from three independent transfections.

## SUPPLEMENTARY MATERIAL FIGURES AND TABLE





## References

[1] Suzuki H, Maruyama R, Yamamoto E, Kai M (2012). DNA methylation and microRNA dysregulation in cancer. Molecular oncology.

[2] Ambros V (2004). The functions of animal microRNAs. Nature.

[3] Lin PC, Chiu YL, Banerjee S, Park K, Mosquera JM, Giannopoulou E, Alves P, Tewari AK, Gerstein MB, Beltran H, Melnick AM, Elemento O, Demichelis F, Rubin MA (2013). Epigenetic repression of miR-31 disrupts androgen receptor homeostasis and contributes to prostate cancer progression. Cancer research.

[4] Althoff K, Beckers A, Odersky A, Mestdagh P, Koster J, Bray IM, Bryan K, Vandesompele J, Speleman F, Stallings RL, Schramm A, Eggert A, Sprussel A, Schulte JH (2013). MiR-137 functions as a tumor suppressor in neuroblastoma by downregulating KDM1A. International journal of cancer Journal international du cancer.

[5] Chen L, Wang X, Wang H, Li Y, Yan W, Han L, Zhang K, Zhang J, Wang Y, Feng Y, Pu P, Jiang T, Kang C, Jiang C (2012). miR-137 is frequently down-regulated in glioblastoma and is a negative regulator of Cox-2. Eur J Cancer.

[6] Balaguer F, Link A, Lozano JJ, Cuatrecasas M, Nagasaka T, Boland CR, Goel A (2010). Epigenetic silencing of miR-137 is an early event in colorectal carcinogenesis. Cancer research.

[7] Liu M, Lang N, Qiu M, Xu F, Li Q, Tang Q, Chen J, Chen X, Zhang S, Liu Z, Zhou J, Zhu Y, Deng Y, Zheng Y, Bi F (2011). miR-137 targets Cdc42 expression, induces cell cycle G1 arrest and inhibits invasion in colorectal cancer cells. International journal of cancer Journal international du cancer.

[8] Vrba L, Munoz-Rodriguez JL, Stampfer MR, Futscher BW (2013). miRNA gene promoters are frequent targets of aberrant DNA methylation in human breast cancer. PloS one.

[9] Tu HF, Lin SC, Chang KW (2013). MicroRNA aberrances in head and neck cancer: pathogenetic and clinical significance. Current opinion in otolaryngology & head and neck surgery.

[10] Langevin SM, Stone RA, Bunker CH, Lyons-Weiler MA, LaFramboise WA, Kelly L, Seethala RR, Grandis JR, Sobol RW, Taioli E (2011). MicroRNA-137 promoter methylation is associated with poorer overall survival in patients with squamous cell carcinoma of the head and neck. Cancer.

[11] Shimizu T, Suzuki H, Nojima M, Kitamura H, Yamamoto E, Maruyama R, Ashida M, Hatahira T, Kai M, Masumori N, Tokino T, Imai K, Tsukamoto T, Toyota M (2013). Methylation of a panel of microRNA genes is a novel biomarker for detection of bladder cancer. European urology.

[12] Zhao Y, Li Y, Lou G, Zhao L, Xu Z, Zhang Y, He F (2012). MiR-137 targets estrogen-related receptor alpha and impairs the proliferative and migratory capacity of breast cancer cells. PloS one.

[13] Eedunuri VK, Rajapakshe K, Fiskus W, Geng C, Chew SA, Foley C, Shah SS, Shou J, Mohamed JS, Coarfa C, O'Malley BW, Mitsiades N (2015). miR-137 Targets p160 Steroid Receptor Coactivators SRC1, SRC2, and SRC3 and Inhibits Cell Proliferation. Mol Endocrinol.

[14] Kashyap V, Ahmad S, Nilsson EM, Helczynski L, Kenna S, Persson JL, Gudas LJ, Mongan NP (2013). The lysine specific demethylase-1 (LSD1/KDM1A) regulates VEGF-A expression in prostate cancer. Molecular oncology.

[15] Wissmann M, Yin N, Muller JM, Greschik H, Fodor BD, Jenuwein T, Vogler C, Schneider R, Gunther T, Buettner R, Metzger E, Schule R (2007). Cooperative demethylation by JMJD2C and LSD1 promotes androgen receptor-dependent gene expression. Nature cell biology.

[16] Metzger E, Wissmann M, Yin N, Muller JM, Schneider R, Peters AH, Gunther T, Buettner R, Schule R (2005). LSD1 demethylates repressive histone marks to promote androgen-receptor-dependent transcription. Nature.

[17] Kahl P, Gullotti L, Heukamp LC, Wolf S, Friedrichs N, Vorreuther R, Solleder G, Bastian PJ, Ellinger J, Metzger E, Schule R, Buettner R (2006). Androgen receptor coactivators lysine-specific histone demethylase 1 and four and a half LIM domain protein 2 predict risk of prostate cancer recurrence. Cancer research.

[18] Mongan NP, Tadokoro-Cuccaro R, Bunch T, Hughes IA (2015). Androgen insensitivity syndrome. Best practice & research Clinical endocrinology & metabolism.

[19] Wang Q, Li W, Zhang Y, Yuan X, Xu K, Yu J, Chen Z, Beroukhim R, Wang H, Lupien M, Wu T, Regan MM, Meyer CA, Carroll JS, Manrai AK, Janne OA (2009). Androgen receptor regulates a distinct transcription program in androgen-independent prostate cancer. Cell.

[20] Kauffman EC, Robinson BD, Downes MJ, Powell LG, Lee MM, Scherr DS, Gudas LJ, Mongan NP (2011). Role of androgen receptor and associated lysine-demethylase coregulators, LSD1 and JMJD2A, in localized and advanced human bladder cancer. Molecular carcinogenesis.

[21] Millard CJ, Watson PJ, Fairall L, Schwabe JW (2013). An evolving understanding of nuclear receptor coregulator proteins. Journal of molecular endocrinology.

[22] Kooistra SM, Helin K (2012). Molecular mechanisms and potential functions of histone demethylases. Nature reviews Molecular cell biology.

[23] Lonard DM, O'Malley BW (2012). Nuclear receptor coregulators: modulators of pathology and therapeutic targets. Nature reviews Endocrinology.

[24] Perillo B, Ombra MN, Bertoni A, Cuozzo C, Sacchetti S, Sasso A, Chiariotti L, Malorni A, Abbondanza C, Avvedimento EV (2008). DNA oxidation as triggered by H3K9me2 demethylation drives estrogen-induced gene expression. Science.

[25] Jin F, Irshad S, Yu W, Belakavadi M, Chekmareva M, Ittmann MM, Abate-Shen C, Fondell JD (2013). ERK and AKT signaling drive MED1 overexpression in prostate cancer in association with elevated proliferation and tumorigenicity. Molecular cancer research : MCR.

[26] Bouchal J, Santer FR, Hoschele PP, Tomastikova E, Neuwirt H, Culig Z (2011). Transcriptional coactivators p300 and CBP stimulate estrogen receptor-beta signaling and regulate cellular events in prostate cancer. The Prostate.

[27] Geng C, He B, Xu L, Barbieri CE, Eedunuri VK, Chew SA, Zimmermann M, Bond R, Shou J, Li C, Blattner M, Lonard DM, Demichelis F, Coarfa C, Rubin MA, Zhou P (2013). Prostate cancer-associated mutations in speckle-type POZ protein (SPOP) regulate steroid receptor coactivator 3 protein turnover. Proceedings of the National Academy of Sciences of the United States of America.

[28] Zhou XE, Suino-Powell KM, Li J, He Y, Mackeigan JP, Melcher K, Yong EL, Xu HE (2010). Identification of SRC3/AIB1 as a preferred coactivator for hormone-activated androgen receptor. The Journal of biological chemistry.

[29] Frescas D, Guardavaccaro D, Kuchay SM, Kato H, Poleshko A, Basrur V, Elenitoba-Johnson KS, Katz RA, Pagano M (2008). KDM2A represses transcription of centromeric satellite repeats and maintains the heterochromatic state. Cell Cycle.

[30] Xiang Y, Zhu Z, Han G, Ye X, Xu B, Peng Z, Ma Y, Yu Y, Lin H, Chen AP, Chen CD (2007). JARID1B is a histone H3 lysine 4 demethylase up-regulated in prostate cancer. Proceedings of the National Academy of Sciences of the United States of America.

[31] Varambally S, Cao Q, Mani RS, Shankar S, Wang X, Ateeq B, Laxman B, Cao X, Jing X, Ramnarayanan K, Brenner JC, Yu J, Kim JH, Han B, Tan P, Kumar-Sinha C (2008). Genomic loss of microRNA-101 leads to overexpression of histone methyltransferase EZH2 in cancer. Science.

[32] Krek A, Grun D, Poy MN, Wolf R, Rosenberg L, Epstein EJ, MacMenamin P, da Piedade I, Gunsalus KC, Stoffel M, Rajewsky N (2005). Combinatorial microRNA target predictions. Nature genetics.

[33] Lewis BP, Burge CB, Bartel DP (2005). Conserved seed pairing, often flanked by adenosines, indicates that thousands of human genes are microRNA targets. Cell.

[34] Wang X (2008). miRDB: a microRNA target prediction and functional annotation database with a wiki interface. RNA.

[35] John B, Enright AJ, Aravin A, Tuschl T, Sander C, Marks DS (2004). Human MicroRNA targets. PLoS biology.

[36] Huang WY, Hsu SD, Huang HY, Sun YM, Chou CH, Weng SL, Huang HD (2015). MethHC: a database of DNA methylation and gene expression in human cancer. Nucleic acids research.

[37] Long Q, Johnson BA, Osunkoya AO, Lai YH, Zhou W, Abramovitz M, Xia M, Bouzyk MB, Nam RK, Sugar L, Stanimirovic A, Williams DJ, Leyland-Jones BR, Seth AK, Petros JA, Moreno CS (2011). Protein-coding and microRNA biomarkers of recurrence of prostate cancer following radical prostatectomy. The American journal of pathology.

[38] Kozaki K, Imoto I, Mogi S, Omura K, Inazawa J (2008). Exploration of tumor-suppressive microRNAs silenced by DNA hypermethylation in oral cancer. Cancer research.

[39] Rehmsmeier M, Steffen P, Hochsmann M, Giegerich R (2004). Fast and effective prediction of microRNA/target duplexes. RNA.

[40] Robinson JT, Thorvaldsdottir H, Winckler W, Guttman M, Lander ES, Getz G, Mesirov JP (2011). Integrative genomics viewer. Nature biotechnology.

[41] Wyce A, Bai Y, Nagpal S, Thompson CC (2010). Research Resource: The androgen receptor modulates expression of genes with critical roles in muscle development and function. Mol Endocrinol.

[42] Cai C, He HH, Gao S, Chen S, Yu Z, Gao Y, Chen MW, Zhang J, Ahmed M, Wang Y, Metzger E, Schule R, Liu XS, Brown M, Balk SP (2014). Lysine-specific demethylase 1 has dual functions as a major regulator of androgen receptor transcriptional activity. Cell reports.

[43] Willmann D, Lim S, Wetzel S, Metzger E, Jandausch A, Wilk W, Jung M, Forne I, Imhof A, Janzer A, Kirfel J, Waldmann H, Schule R, Buettner R (2012). Impairment of prostate cancer cell growth by a selective and reversible lysine-specific demethylase 1 inhibitor. International journal of cancer Journal international du cancer.

[44] Rane JK, Scaravilli M, Ylipaa A, Pellacani D, Mann VM, Simms MS, Nykter M, Collins AT, Visakorpi T, Maitland NJ (2015). MicroRNA expression profile of primary prostate cancer stem cells as a source of biomarkers and therapeutic targets. European urology.

[45] Sun G, Ye P, Murai K, Lang MF, Li S, Zhang H, Li W, Fu C, Yin J, Wang A, Ma X, Shi Y (2011). miR-137 forms a regulatory loop with nuclear receptor TLX and LSD1 in neural stem cells. Nature communications.

[46] Takwi AA, Wang YM, Wu J, Michaelis M, Cinatl J, Chen T (2014). miR-137 regulates the constitutive androstane receptor and modulates doxorubicin sensitivity in parental and doxorubicin-resistant neuroblastoma cells. Oncogene.

[47] Zhu X, Li Y, Shen H, Li H, Long L, Hui L, Xu W (2013). miR-137 inhibits the proliferation of lung cancer cells by targeting Cdc42 and Cdk6. FEBS letters.

[48] Smrt RD, Szulwach KE, Pfeiffer RL, Li X, Guo W, Pathania M, Teng ZQ, Luo Y, Peng J, Bordey A, Jin P, Zhao X (2010). MicroRNA miR-137 regulates neuronal maturation by targeting ubiquitin ligase mind bomb-1. Stem Cells.

[49] Chen DL, Wang DS, Wu WJ, Zeng ZL, Luo HY, Qiu MZ, Ren C, Zhang DS, Wang ZQ, Wang FH, Li YH, Kang TB, Xu RH (2013). Overexpression of paxillin induced by miR-137 suppression promotes tumor progression and metastasis in colorectal cancer. Carcinogenesis.

[50] Li KK, Yang L, Pang JC, Chan AK, Zhou L, Mao Y, Wang Y, Lau KM, Poon WS, Shi Z, Ng HK (2013). MIR-137 suppresses growth and invasion, is downregulated in oligodendroglial tumors and targets CSE1L. Brain Pathol.

[51] Bier A, Giladi N, Kronfeld N, Lee HK, Cazacu S, Finniss S, Xiang C, Poisson L, deCarvalho AC, Slavin S, Jacoby E, Yalon M, Toren A, Mikkelsen T, Brodie C (2013). MicroRNA-137 is downregulated in glioblastoma and inhibits the stemness of glioma stem cells by targeting RTVP-1. Oncotarget.

[52] Sun J, Zheng G, Gu Z, Guo Z (2015). MiR-137 inhibits proliferation and angiogenesis of human glioblastoma cells by targeting EZH2. Journal of neuro-oncology.

[53] Deng Y, Deng H, Bi F, Liu J, Bemis LT, Norris D, Wang XJ, Zhang Q (2011). MicroRNA-137 targets carboxyl-terminal binding protein 1 in melanoma cell lines. International journal of biological sciences.

[54] Langevin SM, Stone RA, Bunker CH, Grandis JR, Sobol RW, Taioli E (2010). MicroRNA-137 promoter methylation in oral rinses from patients with squamous cell carcinoma of the head and neck is associated with gender and body mass index. Carcinogenesis.

[55] Zhang B, Liu T, Wu T, Wang Z, Rao Z, Gao J (2015). microRNA-137 functions as a tumor suppressor in human non-small cell lung cancer by targeting SLC22A18. International journal of biological macromolecules.

[56] Cheng Y, Li Y, Liu D, Zhang R, Zhang J (2014). miR-137 effects on gastric carcinogenesis are mediated by targeting Cox-2-activated PI3K/AKT signaling pathway. FEBS letters.

[57] Cerami E, Gao J, Dogrusoz U, Gross BE, Sumer SO, Aksoy BA, Jacobsen A, Byrne CJ, Heuer ML, Larsson E, Antipin Y, Reva B, Goldberg AP, Sander C, Schultz N (2012). The cBio cancer genomics portal: an open platform for exploring multidimensional cancer genomics data. Cancer discovery.

[58] Li P, Ma L, Zhang Y, Ji F, Jin F (2014). MicroRNA-137 down-regulates KIT and inhibits small cell lung cancer cell proliferation. Biomedicine & pharmacotherapy = Biomedecine & pharmacotherapie.

[59] Marcinkiewicz K, Scotland KB, Boorjian SA, Nilsson EM, Persson JL, Abrahamsson PA, Allegrucci C, Hughes IA, Gudas LJ, Mongan NP (2012). The androgen receptor and stem cell pathways in prostate and bladder cancers (review). International journal of oncology.

[60] de Brot S, Ntekim A, Cardenas R, James V, Allegrucci C, Heery DM, Bates DO, Odum N, Persson JL, Mongan NP (2015). Regulation of vascular endothelial growth factor in prostate cancer. Endocrine-related cancer.

[61] Sharma NL, Massie CE, Ramos-Montoya A, Zecchini V, Scott HE, Lamb AD, MacArthur S, Stark R, Warren AY, Mills IG, Neal DE (2013). The androgen receptor induces a distinct transcriptional program in castration-resistant prostate cancer in man. Cancer cell.

[62] Robinson D, Van Allen EM, Wu YM, Schultz N, Lonigro RJ, Mosquera JM, Montgomery B, Taplin ME, Pritchard CC, Attard G, Beltran H, Abida W, Bradley RK, Vinson J, Cao X, Vats P (2015). Integrative clinical genomics of advanced prostate cancer. Cell.

[63] Culig Z (2015). Androgen Receptor Coactivators in Regulation of Growth and Differentiation in Prostate Cancer. Journal of cellular physiology.

[64] Brooke GN, Parker MG, Bevan CL (2008). Mechanisms of androgen receptor activation in advanced prostate cancer: differential co-activator recruitment and gene expression. Oncogene.

[65] Bevan CL, Hoare S, Claessens F, Heery DM, Parker MG (1999). The AF1 and AF2 domains of the androgen receptor interact with distinct regions of SRC1. Molecular and cellular biology.

[66] Comuzzi B, Nemes C, Schmidt S, Jasarevic Z, Lodde M, Pycha A, Bartsch G, Offner F, Culig Z, Hobisch A (2004). The androgen receptor co-activator CBP is up-regulated following androgen withdrawal and is highly expressed in advanced prostate cancer. The Journal of pathology.

[67] Heemers HV, Sebo TJ, Debes JD, Regan KM, Raclaw KA, Murphy LM, Hobisch A, Culig Z, Tindall DJ (2007). Androgen deprivation increases p300 expression in prostate cancer cells. Cancer research.

[68] Powell SM, Christiaens V, Voulgaraki D, Waxman J, Claessens F, Bevan CL (2004). Mechanisms of androgen receptor signalling via steroid receptor coactivator-1 in prostate. Endocrine-related cancer.

[69] Zhong J, Ding L, Bohrer LR, Pan Y, Liu P, Zhang J, Sebo TJ, Karnes RJ, Tindall DJ, van Deursen J, Huang H (2014). p300 acetyltransferase regulates androgen receptor degradation and PTEN-deficient prostate tumorigenesis. Cancer research.

[70] Liu C, Wang C, Wang K, Liu L, Shen Q, Yan K, Sun X, Chen J, Liu J, Ren H, Liu H, Xu Z, Hu S, Xu D, Fan Y (2013). SMYD3 as an oncogenic driver in prostate cancer by stimulation of androgen receptor transcription. Journal of the National Cancer Institute.

[71] Lee DY, Northrop JP, Kuo MH, Stallcup MR (2006). Histone H3 lysine 9 methyltransferase G9a is a transcriptional coactivator for nuclear receptors. The Journal of biological chemistry.

[72] Shin S, Janknecht R (2007). Activation of androgen receptor by histone demethylases JMJD2A and JMJD2D. Biochemical and biophysical research communications.

[73] Jin F, Claessens F, Fondell JD (2012). Regulation of androgen receptor-dependent transcription by coactivator MED1 is mediated through a newly discovered noncanonical binding motif. The Journal of biological chemistry.

[74] Qin J, Lee HJ, Wu SP, Lin SC, Lanz RB, Creighton CJ, DeMayo FJ, Tsai SY, Tsai MJ (2014). Androgen deprivation-induced NCoA2 promotes metastatic and castration-resistant prostate cancer. The Journal of clinical investigation.

[75] Ianculescu I, Wu DY, Siegmund KD, Stallcup MR (2012). Selective roles for cAMP response element-binding protein binding protein and p300 protein as coregulators for androgen-regulated gene expression in advanced prostate cancer cells. The Journal of biological chemistry.

[76] Bjorkman M, Ostling P, Harma V, Virtanen J, Mpindi JP, Rantala J, Mirtti T, Vesterinen T, Lundin M, Sankila A, Rannikko A, Kaivanto E, Kohonen P, Kallioniemi O, Nees M (2012). Systematic knockdown of epigenetic enzymes identifies a novel histone demethylase PHF8 overexpressed in prostate cancer with an impact on cell proliferation, migration and invasion. Oncogene.

[77] Agoulnik IU, Vaid A, Nakka M, Alvarado M, Bingman WE, Erdem H, Frolov A, Smith CL, Ayala GE, Ittmann MM, Weigel NL (2006). Androgens modulate expression of transcription intermediary factor 2, an androgen receptor coactivator whose expression level correlates with early biochemical recurrence in prostate cancer. Cancer research.

[78] Dasgupta S, Putluri N, Long W, Zhang B, Wang J, Kaushik AK, Arnold JM, Bhowmik SK, Stashi E, Brennan CA, Rajapakshe K, Coarfa C, Mitsiades N, Ittmann MM, Chinnaiyan AM, Sreekumar A (2015). Coactivator SRC-2-dependent metabolic reprogramming mediates prostate cancer survival and metastasis. The Journal of clinical investigation.

[79] Wagner KW, Alam H, Dhar SS, Giri U, Li N, Wei Y, Giri D, Cascone T, Kim JH, Ye Y, Multani AS, Chan CH, Erez B, Saigal B, Chung J, Lin HK (2013). KDM2A promotes lung tumorigenesis by epigenetically enhancing ERK1/2 signaling. The Journal of clinical investigation.

[80] Blackledge NP, Zhou JC, Tolstorukov MY, Farcas AM, Park PJ, Klose RJ (2010). CpG islands recruit a histone H3 lysine 36 demethylase. Molecular cell.

[81] Van Rechem C, Black JC, Greninger P, Zhao Y, Donado C, Burrowes PD, Ladd B, Christiani DC, Benes CH, Whetstine JR (2015). A coding single-nucleotide polymorphism in lysine demethylase KDM4A associates with increased sensitivity to mTOR inhibitors. Cancer discovery.

[82] Casati L, Sendra R, Poletti A, Negri-Cesi P, Celotti F (2013). Androgen receptor activation by polychlorinated biphenyls: epigenetic effects mediated by the histone demethylase Jarid1b. Epigenetics : official journal of the DNA Methylation Society.

[83] Kristensen LH, Nielsen AL, Helgstrand C, Lees M, Cloos P, Kastrup JS, Helin K, Olsen L, Gajhede M (2012). Studies of H3K4me3 demethylation by KDM5B/Jarid1B/PLU1 reveals strong substrate recognition in vitro and identifies 2,4-pyridine-dicarboxylic acid as an in vitro and in cell inhibitor. The FEBS journal.

[84] Taylor BS, Schultz N, Hieronymus H, Gopalan A, Xiao Y, Carver BS, Arora VK, Kaushik P, Cerami E, Reva B, Antipin Y, Mitsiades N, Landers T, Dolgalev I, Major JE, Wilson M (2010). Integrative genomic profiling of human prostate cancer. Cancer cell.

[85] Yamamoto S, Wu Z, Russnes HG, Takagi S, Peluffo G, Vaske C, Zhao X, Moen Vollan HK, Maruyama R, Ekram MB, Sun H, Kim JH, Carver K, Zucca M, Feng J, Almendro V (2014). JARID1B is a luminal lineage-driving oncogene in breast cancer. Cancer cell.

[86] Huang C, Xiang Y, Wang Y, Li X, Xu L, Zhu Z, Zhang T, Zhu Q, Zhang K, Jing N, Chen CD (2010). Dual-specificity histone demethylase KIAA1718 (KDM7A) regulates neural differentiation through FGF4. Cell research.

[87] Yokoyama A, Okuno Y, Chikanishi T, Hashiba W, Sekine H, Fujiki R, Kato S (2010). KIAA1718 is a histone demethylase that erases repressive histone methyl marks. Genes to cells : devoted to molecular & cellular mechanisms.

[88] Arteaga MF, Mikesch JH, Qiu J, Christensen J, Helin K, Kogan SC, Dong S, So CW (2013). The histone demethylase PHF8 governs retinoic acid response in acute promyelocytic leukemia. Cancer cell.

[89] Osawa T, Muramatsu M, Wang F, Tsuchida R, Kodama T, Minami T, Shibuya M (2011). Increased expression of histone demethylase JHDM1D under nutrient starvation suppresses tumor growth via down-regulating angiogenesis. Proceedings of the National Academy of Sciences of the United States of America.

[90] Upadhyay AK, Rotili D, Han JW, Hu R, Chang Y, Labella D, Zhang X, Yoon YS, Mai A, Cheng X (2012). An analog of BIX-01294 selectively inhibits a family of histone H3 lysine 9 Jumonji demethylases. Journal of molecular biology.

[91] Mimasu S, Umezawa N, Sato S, Higuchi T, Umehara T, Yokoyama S (2010). Structurally designed trans-2-phenylcyclopropylamine derivatives potently inhibit histone demethylase LSD1/KDM1. Biochemistry.

[92] Betel D, Wilson M, Gabow A, Marks DS, Sander C (2008). The microRNA. org resource: targets and expression. Nucleic acids research.

[93] Goldman M, Craft B, Swatloski T, Cline M, Morozova O, Diekhans M, Haussler D, Zhu J (2015). The UCSC Cancer Genomics Browser: update 2015. Nucleic acids research.

[94] Mongan NP, Martin KM, Gudas LJ (2006). The Putative Human Stem Cell Marker, Rex-1 (Zfp42): Structural Classification And Expression In Normal Human Epithelial And Carcinoma Cell Cultures. Molecular carcinogenesis.

[95] Huang J, Sengupta R, Espejo AB, Lee MG, Dorsey JA, Richter M, Opravil S, Shiekhattar R, Bedford MT, Jenuwein T, Berger SL (2007). p53 is regulated by the lysine demethylase LSD1. Nature.

[96] Kashyap V, Gudas LJ (2010). Epigenetic regulatory mechanisms distinguish retinoic acid-mediated transcriptional responses in stem cells and fibroblasts. The Journal of biological chemistry.

[97] Mongan NP, Jaaskelainen J, Green K, Schwabe JW, Shimura N, Dattani M, Hughes IA (2002). Two de novo mutations in the AR gene cause the complete androgen insensitivity syndrome in a pair of monozygotic twins. The Journal of clinical endocrinology and metabolism.

