# Supplementary material for: MiR137 is an androgen regulated repressor of an extended network of transcriptional coregulators
Source: Oncotarget. 2015 Oct 5;6(34):35710–25. doi: 10.18632/oncotarget.5958 (PMC4742136; doi:10.18632/oncotarget.5958)
Supplement: Supplementary file 1 [file oncotarget-06-35710-s001.pdf]

MIR137 is an androgen regulated repressor of an extended network of transcriptional coregulators

Supplementary Material

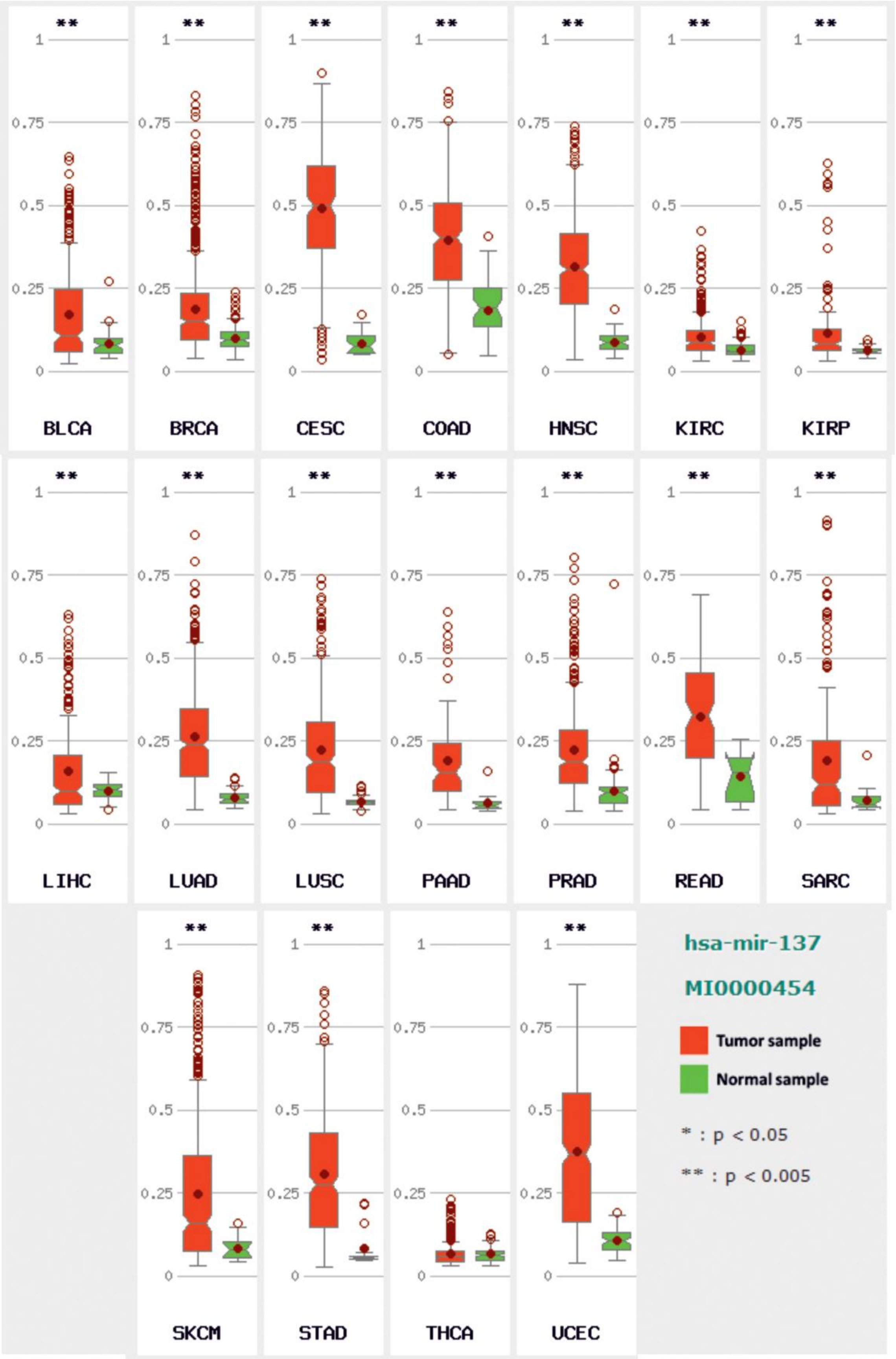
BLCA
BRCA
CESC
COAD
HNSC
KIRC
KIRP
LIHC
LUAD
LUSC
PAAD
PRAD
READ
SARC
SKCM
STAD
THCA
UCEC
hsa-mir-137
MI0000454
Tumor sample
Normal sample
* : p < 0.05
** : p < 0.005

Supplemental Figure 1: DNA methylation status of the *miR137* locus shows significant higher levels of *miR137* locus methylation in diverse cancer types present in the cancer genome atlas including prostate (PRAD), bladder (BLCA), breast (BRCA), cervical (CESC), colon (COAD), head and neck (HNSC), kidney clear cell (KIRC) and papillary (KIRP), liver (LIHC), lung adenocarcimoa (LUAD) and small cell (LUSC), pancreatic (PAAD), rectum (READ), sarcoma (SARC), skin melanoma (SKCM), stomach (STAD) and uterine (UCEC) cancer relative to normal tissue expression (indicated in green). Data was obtained from the methHC dataportal http://methhc.mbc.nctu.edu.tw/php/index.php. Interestingly expression is only unaltered in thyroid carcinoma (THCA).

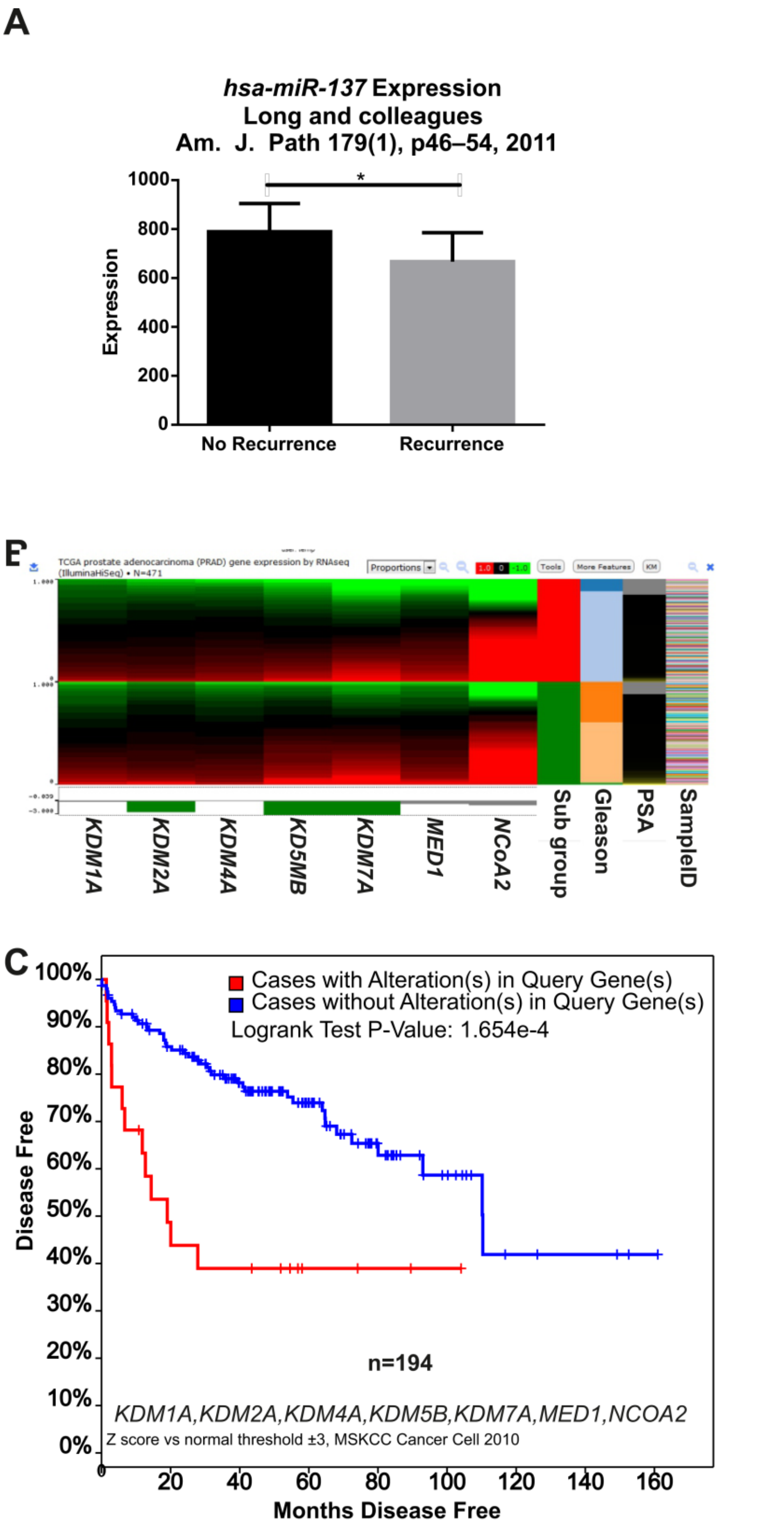

	#total cases	#cases relapsed	Median months disease free
Cases with Alteration(s) in Query Gene(s)	22	13	19.02
Cases wi thout Alteration(s) in Query Gene(s)	152	44	110.33

Supplemental Figure 2. (A) We used a Mann-Whitney test to compare expression of *miR137* expression in a published PCa patient cohort [37]. Median expression of *miR137* was significantly lower in patients who experienced PCa recurrence. **(B)** Using the TCGA PRAD RNAseqV2 dataset, we found that mRNA expression of the novel *miR137* targets identified here correlate with Gleason grade. **(C)** Using the cBio portal we were further able to show that alteration in mRNA expression of *KDM1A, KDM2A, KDM4A, KDM5B, KDM7A, MED1* NCOA2 in primary and metastatic prostate tumors (n=194). Kaplan-Meier analysis indicates expression of these genes is significantly associated with disease free survival in PCa[83].
